# Outcome of teeth restored with CAD/CAM zirconium dioxide post-cores: a retrospective study with a follow-up period of 3–6 years

**DOI:** 10.1186/s12903-022-02273-4

**Published:** 2022-06-16

**Authors:** Shunv Ying, Song Chen, Siyuan Wang, Lingli Xu, Xiaofeng Wang, Fuming He, Wei Liu

**Affiliations:** Stomatology Hospital, School of Stomatology, Zhejiang University School of Medicine, Clinical Research Center for Oral Diseases of Zhejiang Province, Key Laboratory of Oral Biomedical Research of Zhejiang Province, Cancer Center of Zhejiang University, Hangzhou, 310006 China

**Keywords:** Posts, Zirconia, Survival probability, Retrospective study

## Abstract

**Statement of problem:**

Computer aided design/computer aided manufacturing (CAD/CAM) zirconia post-cores is one of the options of post crown restoration materials due to their esthetic properties and superior mechanical strength. However, the clinical effect on aesthetics and strength properties is unclear due to the lack of results of their long-term follow-up.

**Purpose:**

This retrospective clinical study aims to analyze the survival rate, clinical manifestations, and failure factors after CAD/CAM zirconia post-core restoration.

**Material and methods:**

Clinical and radiographic examinations were performed on 342 patients with 400 teeth for 3–6 years postsurgical follow-up examination. The patients were all received CAD/CAM zirconia post-cores and all-ceramic crowns at the Department of Prosthodontics in the public hospital. The retrospective outcomes were conducted after zirconia post restoration, including survival rate by Kaplan–Meier analysis and findings of manifestations and failure factors. The effects of gender and dental position on survival rate were analyzed by Cox–Mantel Test.

**Results:**

This study retrospectively evaluated 261 teeth from 229 patients with a 35% drop-out rate. The survival rate was 96.0%, and the success rate was 92.4%. According to the tooth position classification, the survival rate was 100% for 101 anterior teeth, 95.4% for 69 premolars, and 88.3% for 91 molars. According to gender, the survival rate of the male group was 92.3%, while that of the female group was 98.0%, with a significant difference (*P* < 0.01). The complications included crown fracture (1.9%) periapical inflammation (1.9%), crown debonding (1.1%), percussion abnormal (1.9%) and root fracture (0.8%).

**Conclusions:**

Within the limitations of this retrospective study, it can be concluded that CAD/CAM zirconia post-cores are clinically promising. Compared with the posterior teeth, CAD/CAM zirconia post-cores are more suitable for anterior teeth.

## Introduction

The restoration of structurally compromised teeth usually requires post-core techniques to improve teeth support and retention, especially when the remaining tooth structure is incapable of sustaining the occlusal forces exerted by coronal restorations. These posts may be fabricated using various materials, such as metal, fiber, or zirconia.

The existing evidence suggests that the survival rates of metal ranged between 77.9 and 91.7% and that of fiber posts ranged between 84.7 and 100% over the past decade [1–4]. Sarkis conducted a 3-year retrospective study and found that the metal posts had a 91.9% success rate and the fiber posts had a 97.1% success rate [1]. Kramer et al. followed 140 teeth that required post crown restoration after root canal treatment for 59 months and found that the success rate was 83%, and that the success rate of fiber posts was lower than that of metal post [2]. A recent systematic review revealed a lack of studies comparing cast metal and fiber-glass posts with longer follow-up [5] but suggested that metal posts performed better in teeth with no ferrule.

However, both materials have significant limitations. The high elastic modulus of metal may result in an increased concentration of stress in the surrounding radicular dentin, leading to root fracture. Additional disadvantages include microleakage, corrosion, and compromised esthetic properties caused by metal reflection on all-ceramic restorations [6]. In contrast, fiber-reinforced posts can minimize the risk of root fractures and exhibit significantly higher survival rates. However, the limited diameters of most fiber-post systems are unsuitable for conservative tooth preparation, and the post shape often makes it unsuitable for personalized tooth restoration, especially in teeth with large defects [7].

Nowadays, zirconia posts have become a popular alternative for structurally compromised and root-filled teeth, particularly for patients with esthetic demands. Zirconia, a crystalline dioxide of zirconium, was first used in dentistry by Meyenberg et al. in 1993 [8]. It has been widely used in dental restorations, such as crowns, implants, and fixed partial dentures, due to its incontestably superior characteristics in terms of radio-opacity, esthetics, biocompatibility, and mechanical properties. Moreover, the introduction of computer aided design/computer aided manufacturing (CAD/CAM) in dentistry has expedited the application of zirconia post-cores [9–11]. Various in vitro studies suggests that there are questionable clinical efficacy due to high elastic modulus and low fracture resistance of CAD/CAM zirconia posts compared with dentin [12].

Clinical data on the long-term survival and success probability of CAD/CAM zirconia posts exhibited substantial heterogeneity with the reported survival rates ranging from 81.3 to 100% [13–16]. For example, Zhao et al. [15] conducted a prospective study on 20 post-cores made with CAD/CAM zirconia posts and resin cores and found that the 2-year success rate was 86.1%. Pang [17] conducted a 2-year prospective study on 100 anterior teeth restored with post-cores, made with zirconia posts and resin cores, and found that the success rate was 95.6%. Bateli [16] followed up 64-zirconia precast and resin-core Cosmopost for 12 years with a success rate of 81.3%. However, most post-cores used in these studies were zirconia posts combined with other materials, such as composite resin core. The inclusion criteria and follow-up time of these studies were also different. Different clinical procedures, follow-up periods, and bonding protocols may lead to inconsistent reporting of clinical outcomes that were further aggravated by limited sample sizes and high withdrawal rates. Additionally, medium- and long-term clinical data on the CAD/CAM integrated zirconia post-core are currently still lacking. Studies with reasonable follow-up time are useful, especially with more predictable reparative therapy, as some materials may fail late, and studies with follow-up periods < 2–3 years currently have limited clinical relevance [18, 19]. Therefore, a large-scale clinical study on CAD/CAM integrated zirconia post-core using standardized protocols following the same procedure is necessary.

In this study, 400 compromised teeth underwent CAD/CAM zirconia post-core restoration performed by two experienced operators between July 2014 and December 2017. The patients were followed up for 3–6 years to record their complications, including root fracture, post fracture, periapical inflammation, impaired periodontal health, delamination or debonding of the restoration, and patient’s perceptions. Based on this the long-term clinical performance of CAD/CAM zirconia posts were evaluated and the potential limitations were identified.

## Material and methods

### Patient selection

This study was approved by the bioethics committee of the School of Stomatology of Zhejiang University (202134). The research team therefore had access to patients' electronic records. One blinded senior examiner identified patients with teeth that had received CAD/CAM zirconia post-cores by screening the patient archives at the Department of Prosthodontics. The patients had volunteered and provided signed informed consent for dental prostheses consisting of integrated CAD/CAM zirconia post-cores and all-ceramic crowns.

The case inclusion criteria were as follows: (a) no loosening of affected teeth; (b) completed root canal treatment; (c) no obvious clinical symptoms; (d) no periodontal inflammation. While the patient exclusion criteria were as follows: (a) patients that were seriously ill, had mental illnesses, incapacitated, lliterate, or mental retardation, etc.; (b) prisoners, children, and pregnant women; (c) smokers; (d) patients < 18 years of age at the time of the first visit.

The final study samples consisted of 400 endodontically-treated teeth in 342 patients which were retrospectively evaluated. Periodontal treatment, root canal treatment, and occlusal adjustments were conducted when necessary before prosthetic treatment. All preparations were made in accordance with unified process to minimize the study's heterogeneity and increase reliability by using CAD/CAM zirconia post-cores from the same manufacturer (Dentsply, Sirona, Germany) and restricting two senior operators.

### Prosthetic protocol

Before restoration, the root canal fillings were evaluated radiographically with regard to length, density, adaptation to the root canals, and periapical conditions. All teeth were prepared for post restorations in accordance with unified operation method. We determined the working length according to the root canal length and marked it on the reamer. Then, we drilled in at low speed in the direction of the root canal and pulled the tape out until the desired working length. According to the length, shape and diameter of the root of the affected tooth, the corresponding type of P drill was used as the final drill needle for preparation to the predetermined working length. The post was longer than the height of the clinical crown and retained 5 mm of canal filling at least at the apex. A ferrule with a minimum height of 1.5 mm was also prepared consistent with the residual hard tissue.

**Fig. 1:**
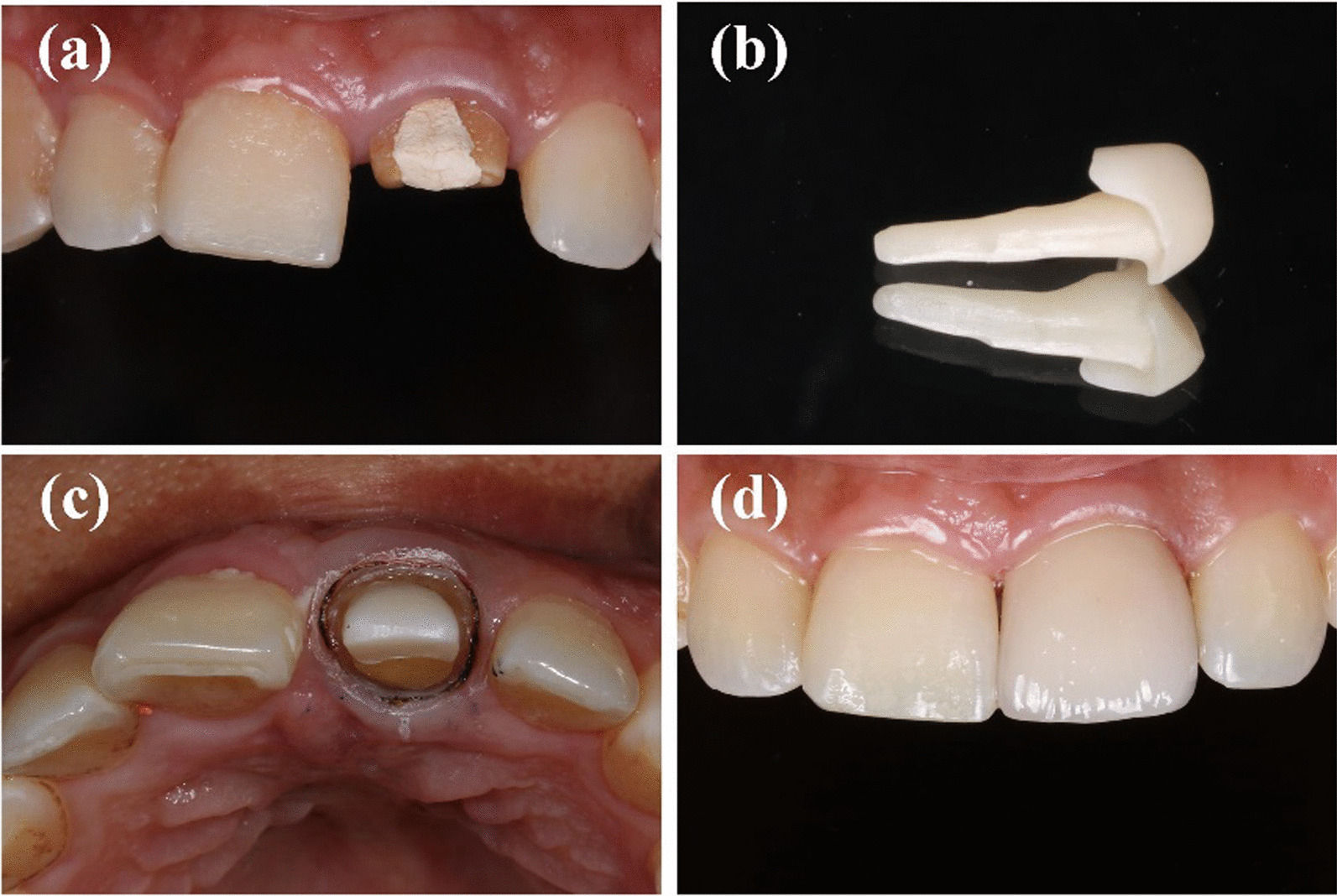
Manufacture process of CAD/CAM zirconia post for anterior teeth for anterior teeth

**Fig. 2:**
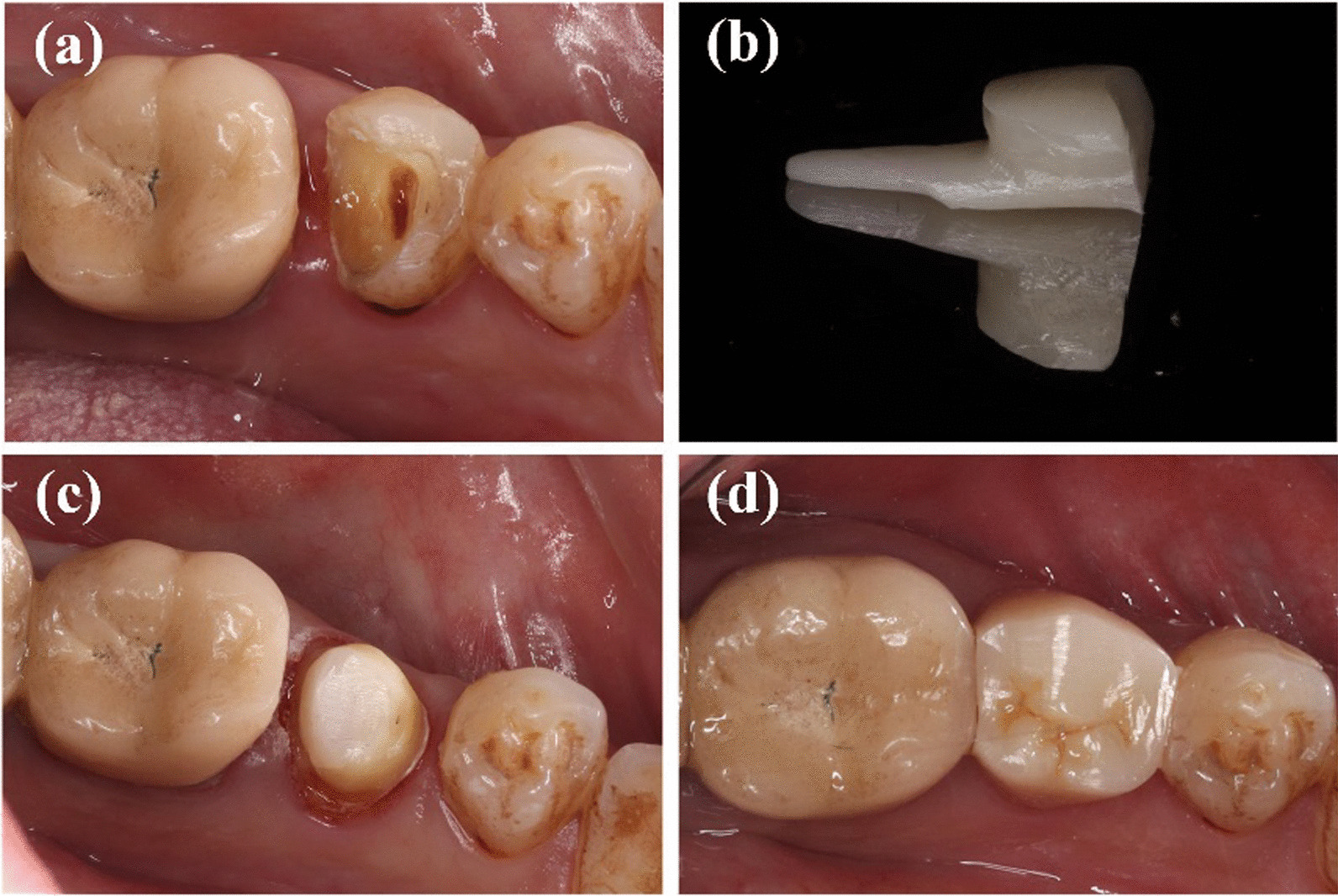
Manufacture process of CAD/CAM zirconia post for premolar

**Fig. 3:**
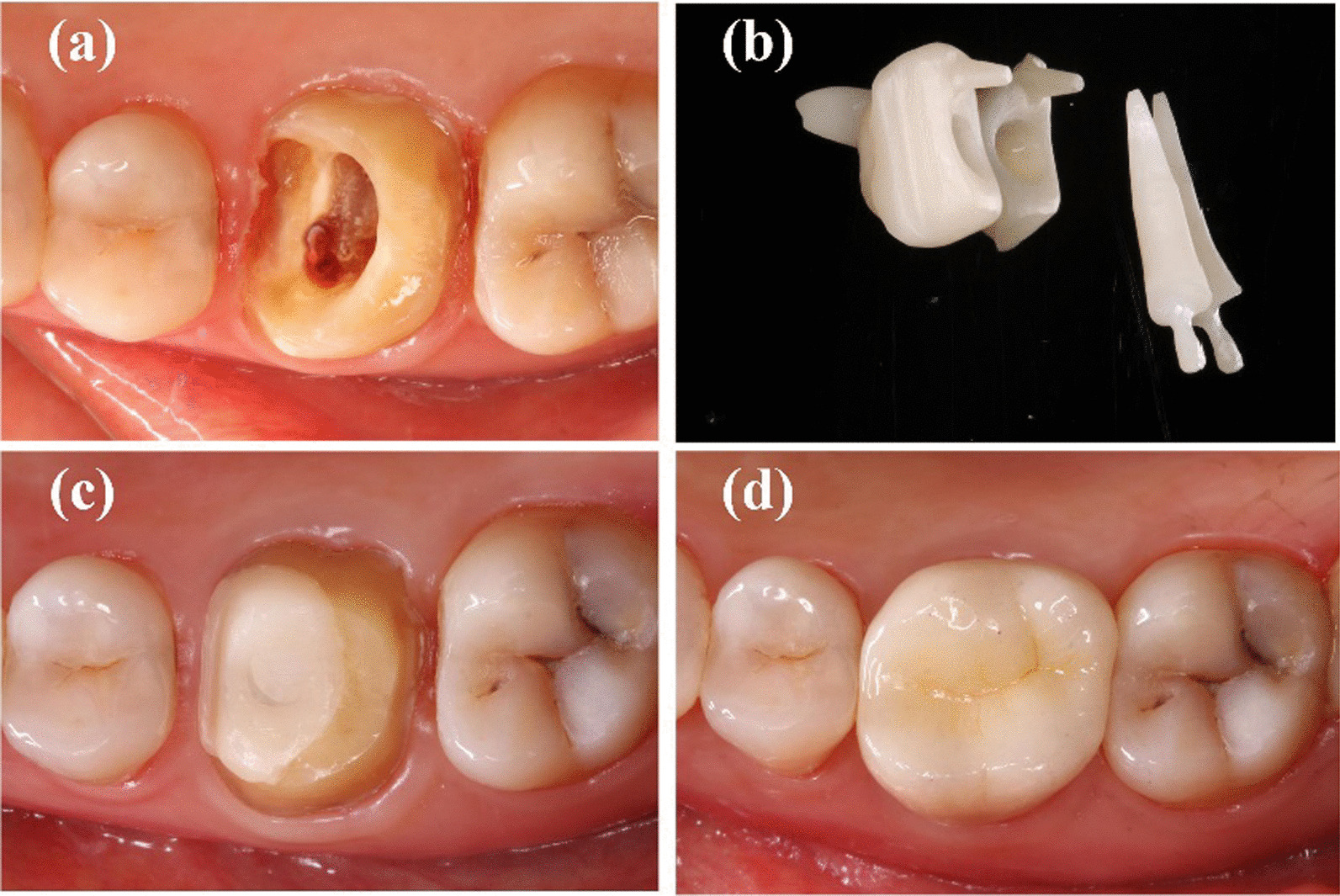
Manufacture process of CAD/CAM zirconia post for molar

Thereafter, impressions were made using polysiloxane impression material (3 M, Maplewood, MN, USA) and a type wax was created and scanned after spraying scanning powder (DS-EX Proceed; Shining 3D, Zhejiang, China). The deputy cast post was designed using the CAD/CAM system (Ceramill; Amann Girrbach, Charlotte, NC, USA), and the zirconium oxide block (Dentsply, Sirona, Germany) was cut with a grinding apparatus (Ceramill Motion 2, Amann Girrbach). Finally, a sintering furnace (Infire HTC speed; Dentsply Sirona, York, PA, USA) was used. All post-cores were polished and cemented conventionally using resin cement (RelyX™ Unicem, 3M,US). After curing, dental preparation and conventional crown restoration were performed. All affected teeth in this study were restored using zirconia all-porcelain crowns made by CAD/CAM integration (Dentsply, Sirona, Germany and 3M, Lava, USA.). All crowns were sand-blasted and cemented conventionally using resin cement (RelyX™ Unicem, 3M, US). The production process with different tooth positions has been shown in Figs. 1, 2 and 3.

### Data collection and analysis

The follow-up examinations, conducted by two prosthodontists, consisted of the evaluation of the patients’ perceptions, clinical measurements, and radiographic examinations. The success criteria were established as follows: (1) probing depths ≤ 3 mm at six aspects; (2) lack of mobility of the crown or post; (3) lack of tooth sensitivity to horizontal and vertical percussion; (4) no sign of periapical inflammation; and (5) no breakage of the detachment of the post and crown [20].

Failure cases were divided into catastrophic and noncatastrophic complications. Catastrophic complications were defined as tooth extractions due to root fracture, severe periodontitis, or postfracture. Noncatastrophic complications included percussion sensitivity or other signs of periapical inflammation, fracture of the core or crown, post debonding, and crown dislodgement.

The longevity of the zirconia post was measured up to the day of teeth extraction, catastrophic complications, or clinical examination if no catastrophic complications occurred. Survival rate was assessed using Kaplan–Meier analysis by Graphpad Prism 8.0 and SPSS, and survival percentages were calculated up to 5 years of follow-up due to the small sample size after that. Cases without any complications were considered successful to access success rate, and any differences between tooth positions and gender were examined by Cox–Mantel Test with post hoc contrasts by Bonferroni test. The statistical significance for all tests was set at *P* < 0.05.

## Results

**Fig. 4:**
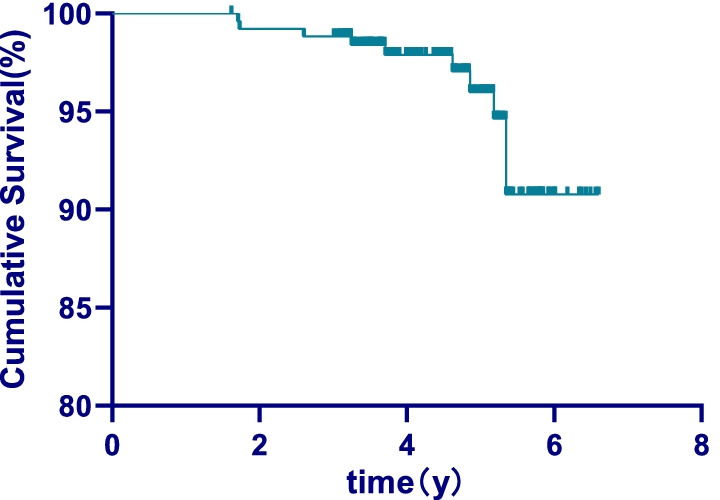
The overall survival curve

**Fig. 5:**
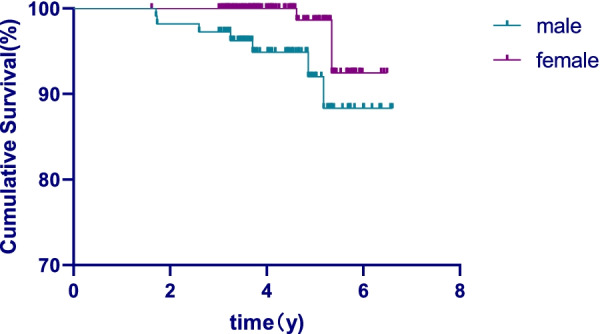
Survival curve in different genders

**Fig. 6:**
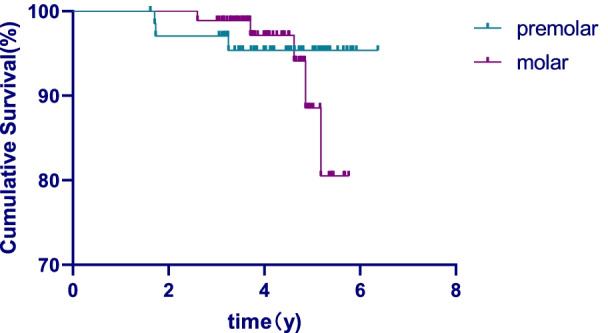
Survival curve in different tooth position

This study retrospectively evaluated 400 teeth from 342 patients. The drop-out rate was 35%, resulting in inclusion of 261 teeth of 229 individuals in the end. The mean age was 37.9 years (range: 18–79 years), and the mean follow-up time was 54.2 months. The overall success rate was 92.3%, including 95.5% for 101 anterior teeth, 91.3% for 69 premolar teeth, and 90.3% for 91 molar teeth. The overall survival rate was 96%. According to the tooth position classification, the survival rate was 100% for anterior teeth, 95.4% for premolars, and 88.3% for molars. The survival rate of the anterior teeth group was significantly higher than that of premolars group and molars group (*P* < 0.05), but there was no significant difference between premolars group and molars group (*P* > 0.05). According to gender, the survival rate of the male group was 92.3% and that of the female group was 98.0%, with a significant difference (*P* < 0.05). The survival curves have been depicted and shown in Figs. 4, 5 and 6.

**Fig. 7:**
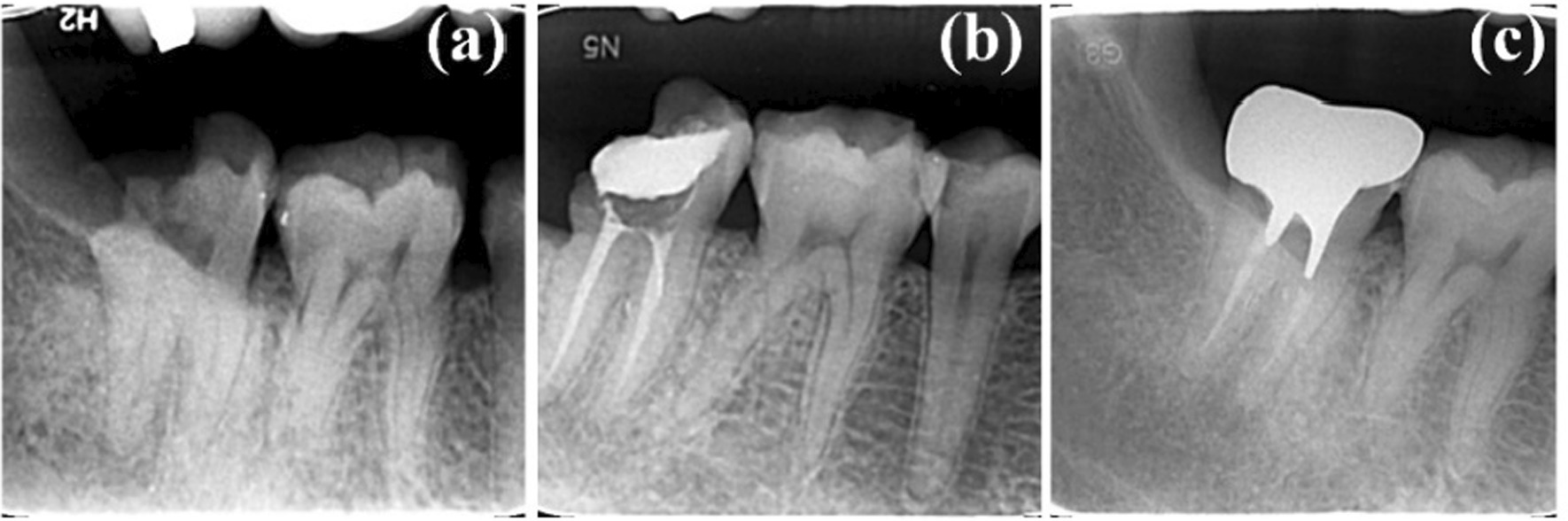
**a** X-rays at initial diagnosis, **b** X-rays after root canal treatment, **c** X-rays of 4 years after the restoration was completed

**Fig. 8:**
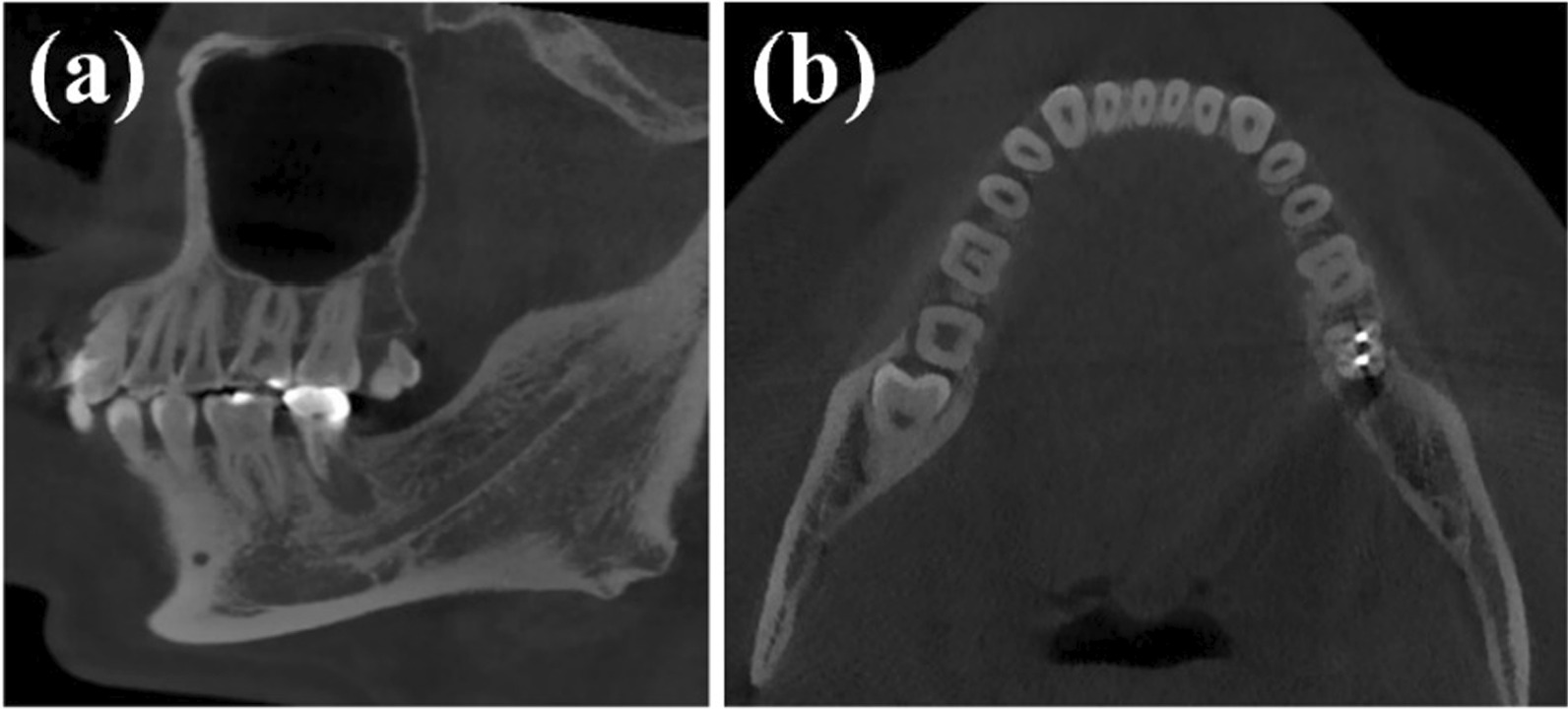
**a** Periapical inflammation in CAD/CAM zirconia post in mandibular molar, **b** root fracture in CAD/CAM zirconia post in mandibular molar

Of the 261 CAD/CAM zirconia post-cores examined, 241 restorations were in clinical use without any signs of failure (Fig. 7). Complications were observed in 20 post-cores at the time of the follow-up examination, and the details of the failures observed have been shown in Table 1. The results showed that six of the affected teeth (premolars and molars) had been removed or were about to be removed for the following reasons: root fracture (two cases, Fig. 8B), severe apical inflammation (three cases, Fig. 8A), and loss cementation (one case). Furthermore, 14 teeth exhibited noncatastrophic complications including crown debonding (two cases), porcelain collapse (five cases), aggravating apical inflammation (two cases), and sensitivity to percussion (five cases).

**Table 1:** Complications in 261 CAD/CAM zirconia posts

Tooth position	Totality	Success rate (%)	Root fracture	Periapical inflammation	Crown fracture	Crown debonding	Percussion abnormal
Anterior teeth	101	95	0	2	0	0	3
Premolar	69	91.3	1	2	3	0	0
Molar	91	90.2	1	1	2	3	2
Total	261	92.4	2	5	5	3	5

## Discussion

The observation time in this study was 3 to 6 years (mean 4.5 years), 229 patients (67.0% of the total number of patients) with 261 posts (65.3% of the total number of posts) participated in the follow-up study. The 35% drop-out rate (32 patients) was due to the following reasons: 26% of the patients were untraceable, and 19% of the patients were reluctant in returning. The drop-out rate was attributable to inconvenient transportation and poor patient compliance and this affected the findings of this study. The results showed that the success rate of CAD/CAM zirconia post-cores was 92.3% after 5 years of observation, whereas the survival rate was 96.0%. The result was higher than that of the previous studies using zirconia posts combined with the resin cores, which was also higher than that of the metal post-cores [1, 2]. In this study, the patients’ gender impacted the longevity of the posts. The success rate in female patients (98%) was higher than in male patients (92.3%) Still, the effects of gender have not been analyzed in the current studies after root canal restoration, the reasons for this result needed to be further studied.

Previous studies revealed that the longevity of posts was significantly influenced by tooth type. Compared with posterior teeth, higher failure rates for restorations of anterior teeth were observed, because anterior teeth suffer from the lateral force [17, 20]. Other studies have found that there is no difference in the success rate between anterior and premolars [2]. This was in contrast to the results of the present study. In this study, survival rate of anterior teeth was significantly higher than that of premolar and molar group, but there was no significant difference between molars and premolars group. During the follow-up period, two cases of anterior teeth appeared severe periapical inflammation, apical surgery was considered as a further treatment plan. But when it happened to molar, patients and doctors choosed to tooth extraction because of poor surgical field, high surgical difficulty, and uncertain prognosis. In addition, the affected teeth were fully grinded to reduce the interference of anterior extension, and reduce the lateral force on the anterior teeth. However, there were too few failed cases in this study, which had certain limitations. Longer follow-up will be required to obtain long-term results. The rigidity of post materials is one of the important factors in material selection. Excessive rigidity will tend to transfer inconsistent stresses to the compromised tooth, thus, increasing the risk of root fracture [21]. Habibzedha et al. reported significantly lower fracture resistance in mandibular premolars restored with one-piece custom-milled zirconia posts compared with cast Ni–Cr post and prefabricated fiber-glass posts with composite resin cores [7]. Data from the retrospective study shows that the probability of root fracture within 3 years of zirconia post restoration was 1.19% [20]. In this study, tooth extraction due to root fracture was observed in one molar (Fig. 8A) and one premolar after 6 years of follow-up. The probability of root fracture was 0.08%, and it was likely due to the strict inclusion criteria. Both affected teeth had a ferrule of 1.5 mm. Dong Chen et al. measured the stress distribution of a maxillary canine restored by using zirconia posts with different ferrule heights and found that dentinal ferrules of a sufficient height shifted the stress concentration coronally. Thus, it not only eased the stress at the apical root and postdentin interface but also attenuated the wedging force of posts against the root structures [22–24]. The thickness of the binder between the zirconia post and root canal wall was also thought to act as a buffer, although further tests are necessary to confirm this.

Three cases exhibited severe apical inflammation during the follow-up period and were at a higher potential of requiring extraction. Although apical radiographs before repair showed that all teeth had some degree of apical inflammation at the time of initial diagnosis, follow-up radiographs showed expansion of the inflamed area and absorption of the apical bone (Fig. 8B), suggesting the failure of root canal therapy. However, it is unclear whether this could be attributed to the zirconia post-cores. Two cases of anterior teeth exhibited slightly increased apical inflammation without obvious clinical symptoms, and apical surgery was considered as a further treatment plan. Moreover, five cases presented with an abnormal response to percussion and self-reported occlusal discomfort, although no occlusal interference, root fracture, or recurrence of apical inflammation was observed. Further follow-up and observation were recommended by the surgeon.

Shin et al. found that zirconia alone or blocks cemented with resin-modified glass-ionomer cement demonstrated acceptable biocompatibility, although a slight increase of inflammatory cytokines was also observed in their in vitro studies assessing the cytotoxic effects and biocompatibility of zirconia post-cores. The addition of surface modifications to zirconia posts was also found to increase their biocompatibility [25, 26] and further investigation of the role of cements on subsequent periapical inflammation in patients with CAD/CAM zirconia post restorations is necessary. In this study, three cases of dental crown shedding in the molar area were observed, but there were no loose core shedding of CAD/CAM zirconia post-cores. The incidence of crown shedding was 1.9%, which is consistent with previous studies [17]. Moreover, previous studies have also shown acceptable adhesion between the zirconia post and tooth [27]. In this study, five cases (two premolars and three molars) exhibited crown collapse during the follow-up period, but no post rupture was observed. The probability of crown collapse was 1.9%, which is a common complication of zirconia all-ceramic crowns, consistent with the rates of porcelain collapse reported in previous studies [9, 28]. Regrettably, abnormal occlusal function was not recorded at follow-up and thus was not described in the results. No significant parafunctions were found in the analysis of cases of crown collapse. Among the cases of crown fracture, there were two patients with enamel attrition on the occlusal surface, but the history of night bruxism and clenching was denied. There were too few cases of failure, so statistical results could not be obtained, but it suggested that patients with large bite force were prone to crown porcelain breakage. In future studies, we will add occlusal function to follow-up indicators to explore the long-term impact of abnormal occlusal function on the prosthesis. All affected teeth in this study were restored using zirconia post-cores and zirconia all-porcelain crowns made by CAD/CAM integration. There were no patient complaints with the appearance of the restorations due to the good esthetic properties of zirconia material. Generally, CAD/CAM zirconia post-cores can be recommended when the use of a prefabricated post is not suitable due to the anatomy of the root canal, extensive loss of the coronal tooth portion, high esthetic demands, and ceramic crown thickness less than 1.6 mm [29, 30]. Overall, CAD/CAM zirconia post-cores offer a unique advantage in esthetically-demanding cases.

In addition, this retrospective study was conducted over 3–6 years, and a longer follow-up period would allow the determination of the long-term clinical effects of CAD/CAM zirconia post-cores.

## Conclusions

Within the limitations of this retrospective study, it can be concluded that CAD/CAM zirconia post-cores have good long-term clinical performance, with an overall success rate of 92.3%. 6 teeth had been failure for the reasons as root fracture, severe apical inflammation, and loss cementation. Furthermore, 14 teeth exhibited noncatastrophic complications including crown debonding, porcelain collapse, aggravating apical inflammation, and sensitivity to percussion. Therefore, we may safely conclude that the clinical applications of CAD/CAM zirconia post-cores are promising. In the selection of post-cores materials, CAD/CAM zirconia post-cores may be a reliable treatment option.

## Data Availability

The datasets used and/or analysed during the current study are available from the corresponding author on reasonable request.
